# The role of the pre-commissural fornix in episodic autobiographical memory and simulation

**DOI:** 10.1016/j.neuropsychologia.2020.107457

**Published:** 2020-05

**Authors:** Angharad N. Williams, Samuel Ridgeway, Mark Postans, Kim S. Graham, Andrew D. Lawrence, Carl J. Hodgetts

**Affiliations:** a Cardiff University Brain Research Imaging Centre (CUBRIC), School of Psychology, Cardiff University, Maindy Road, Cardiff, CF24 4HQ, United Kingdom; b Max Planck Research Group Adaptive Memory, Max Planck Institute for Human Cognitive and Brain Sciences, Stephanstraße 1a, 04103, Leipzig, Germany; c Department of Psychology, Royal Holloway, University of London, Egham, Surrey, TW20 0EX, United Kingdom

**Keywords:** Hippocampus, Episodic memory, Future thinking, Mental time travel, vmPFC, White matter tractography

## Abstract

Neuropsychological and functional magnetic resonance imaging evidence suggests that the ability to vividly remember our personal past, and imagine future scenarios, involves two closely connected regions: the hippocampus and ventromedial prefrontal cortex (vmPFC). Despite evidence of a direct anatomical connection from hippocampus to vmPFC, it is unknown whether hippocampal-vmPFC structural connectivity supports both past- and future-oriented episodic thinking. To address this, we applied a novel deterministic tractography protocol to diffusion-weighted magnetic resonance imaging (dMRI) data from a group of healthy young adult humans who undertook an adapted past-future autobiographical interview (portions of this data were published in Hodgetts et al., 2017a). This tractography protocol enabled distinct subdivisions of the fornix, detected previously in axonal tracer studies, to be reconstructed *in vivo*, namely the pre-commissural (connecting the hippocampus to vmPFC) and post-commissural (linking the hippocampus and medial diencephalon) fornix. As predicted, we found that inter-individual differences in pre-commissural - but not post-commissural - fornix microstructure (fractional anisotropy) were significantly correlated with the episodic richness of both past *and* future autobiographical narratives. Notably, these results held when controlling for non-episodic narrative content, verbal fluency, and grey matter volumes of the hippocampus and vmPFC. This study provides novel evidence that reconstructing events from one's personal past, and constructing possible future events, involves a distinct, structurally-instantiated hippocampal-vmPFC pathway.

## Highlights

• Hippocampal-vmPFC interactions critical to a number of memory and imagination functions.
• Precommissural fornix provides sole route for direct hippocampal-vmPFC connections.
• Healthy participants completed past-future autobiographical interview and diffusion MRI.
• Precommissural fornix microstructure linked to episodic content of past and future narratives.

## 1 Introduction

A key adaptive feature of human cognition is the ability to re-experience our personal histories and imagine the future in vivid detail (Suddendorf and Corballis, 2007; Tulving, 2005; Wheeler et al., 1997). Building on a key insight from Tulving (1985), according to the constructive episodic simulation hypothesis, the processes and neural machinery that allow us to remember past experiences also allow us to imagine future experiences (Addis, 2018; Schacter et al., 2012). Consistent with this view, remembering past and imagining future events activate a common set of brain regions, including the hippocampus and ventromedial prefrontal cortex (vmPFC) (Addis et al., 2007; Benoit and Schacter, 2015). Furthermore, the ability to retrieve episodically rich autobiographical memories and construct coherent future simulations is diminished following lesions to both the hippocampus and vmPFC (Kwan et al., 2010; McCormick et al., 2018; Race et al., 2011; but see Dede et al., 2016). Such findings have led to the suggestion that the hippocampus and vmPFC are critical nodes within a default (Andrews-Hanna et al., 2010; Raichle, 2015) or ‘core’ network that interact to support autobiographical memory and imagination (Schacter et al., 2012; Schacter et al., 2017; for related proposals see also Buckner and Carroll, 2007; McCormick et al., 2018; Murray et al., 2017; Robin and Moscovitch, 2017; Sheldon and Levine, 2016).

Converging evidence has shifted focus towards this neural network-level approach (Mesulam, 1995; Tulving and Markowitsch, 1997) to support the way we reconstruct our personal past and construct possible future experiences (Bellana et al., 2017; Schacter et al., 2012; Schacter et al., 2017). For instance, studies using functional magnetic resonance imaging (fMRI) have found increased functional connectivity between the hippocampus and vmPFC during both the retrieval of autobiographical memories (McCormick et al., 2015) and the construction of episodic future events (Campbell et al., 2018), and resting-state functional connectivity between these regions has been shown to predict the episodic quality of individual's memories (Yang et al., 2013; see also Miller et al., 2020).

The communication of information across networked areas depends on the organization and integrity of the white matter connections between them (Jbabdi and Behrens, 2013). Invasive tract-tracing techniques have revealed direct efferent anatomical connections from the hippocampus to the vmPFC. In rats, the entire longitudinal extent of the subiculum/CA1 is connected - via the pre-commissural fornix - with the vmPFC, with connectivity increasing progressively in strength from dorsal to ventral hippocampus (Cenquizca and Swanson, 2007; Jay and Witter, 1991). Similarly in primates, the pre-commissural fornix provides the exclusive route for subiculum/CA1 (and possibly CA3) projections to medial and orbital PFC (Aggleton et al., 2015; Barbas and Blatt, 1995; Carmichael and Price, 1995), with relatively more projections arising from the anterior hippocampus. In humans, diffusion-weighted magnetic resonance imaging (dMRI), which can non-invasively delineate the path of major fiber pathways and evaluate their microstructure through indices such as fractional anisotropy (FA) (Jbabdi and Behrens, 2013), has provided initial evidence for hippocampus-PFC connections via the fornix (Croxson et al., 2005). Building on this work, Christiansen et al. (2016) recently developed an anatomically-guided dMRI protocol for the selective *in vivo* reconstruction of pre-commissural fornix fibers in humans, allowing investigation of the functions supported by human hippocampus-10.13039/501100000108PFC direct structural connectivity for the first time.

Through the application of this novel, anatomically-informed tractography protocol, we investigated the role of the pre-commissural fornix in autobiographical past and future thinking using an individual differences design (Palombo et al., 2018b; Tulving et al., 1999). Some of the data from the experiment described below have been reported in a prior publication (Hodgetts et al., 2017a), which examined the relationship between microstructure of the fornix as one unified bundle and episodic versus semantic autobiographical memory. Participants were asked to recall past experiences and generate future events using word-cues according to a modified Galton-Crovitz cue-word paradigm (Crovitz and Schiffman, 1974). White matter microstructure was assessed in these individuals using high angular resolution diffusion-weighted imaging (HARDI) and constrained spherical deconvolution tractography, which permits tracking through regions of crossing fibers (Dell'Acqua and Tournier, 2019). Given the directed hippocampus-PFC functional connections identified above in relation to (re)constructing events in episodic memory and episodic simulation (Campbell et al., 2018; McCormick et al., 2015), we hypothesized that individual differences in the episodic richness of past and future thinking would be related to the microstructure of the hippocampus-PFC connections underpinned by the pre-commissural fornix. As a comparison tract, we used the post-commissural fornix, which connects hippocampus to mammillary bodies and anterior thalamic nuclei (Aggleton, 2012; Christiansen et al., 2016; Mathiasen et al., 2019).

## 2 Materials and methods

### 2.1 Participants

Participants were 27 healthy Cardiff University undergraduates (aged 18–22 years; mean age = 19; 25 females, 2 males). Portions of this data have been published previously (Hodgetts et al., 2017a). Here we address a novel and distinct question, combining our prior autobiographical data with unpublished data from a future thinking task in the same subjects, and a novel anatomically-informed tractography protocol for reconstructing distinct fornix subdivisions. Participants completed an adapted autobiographical past-future cue-word paradigm (Addis et al., 2008; Crovitz and Schiffman, 1974) in a separate session approximately 10 months after the original imaging data acquisition. All participants gave written informed consent before participating. Cardiff University School of Psychology Research Ethics Committee reviewed and approved this research.

## 3 Experimental design

### 3.1 Past-future autobiographical interview (AI) task procedure

Participants completed an adapted autobiographical cue-word paradigm (Addis et al., 2008; Crovitz and Schiffman, 1974) that probed both past and future events. In each of the two conditions (past, future), ten cue-words (e.g. “holiday”, “birthday”) were provided to each participant, in response to which they were asked to recall or imagine a personal event and to generate as much detail as possible within 1-min (see Cole et al., 2012). Each event was required to be spatiotemporally specific, occurring over a timescale of minutes or hours, but no longer than a day. Future events were required to be plausible given the participant's current plans and not previously experienced by the participant. Three alternate word lists were used; these were matched for semantic category (i.e., participants either heard the cue-word ‘holiday’, ‘journey’ or ‘vacation’). Prior to commencing, participants were instructed:

*“In this test I am going to give you a series of words and ask you to recall an episode from your past, or think of an episode that you might be involved in in the future, related to each of these words. The episode needs to be as specific and detailed as possible. I would like you to give me as much information as you can.”*

In cases where the participant either lacked specificity or detail in their description, the experimenter would provide a non-specific prompt for further information (e.g., *“Is there anything else you can tell me about this event?“).* All trials for one temporal direction (past or future) were completed before beginning the trials for the other condition. Order of presentation of temporal direction (past or future) was counterbalanced, as were the word lists (across the past and future conditions). Participants were tested individually, and responses were recorded using a portable recording device (Zoom H1 Digital Field Recorder) for subsequent transcription and scoring.

### 3.2 Scoring

**Fig. 1:**
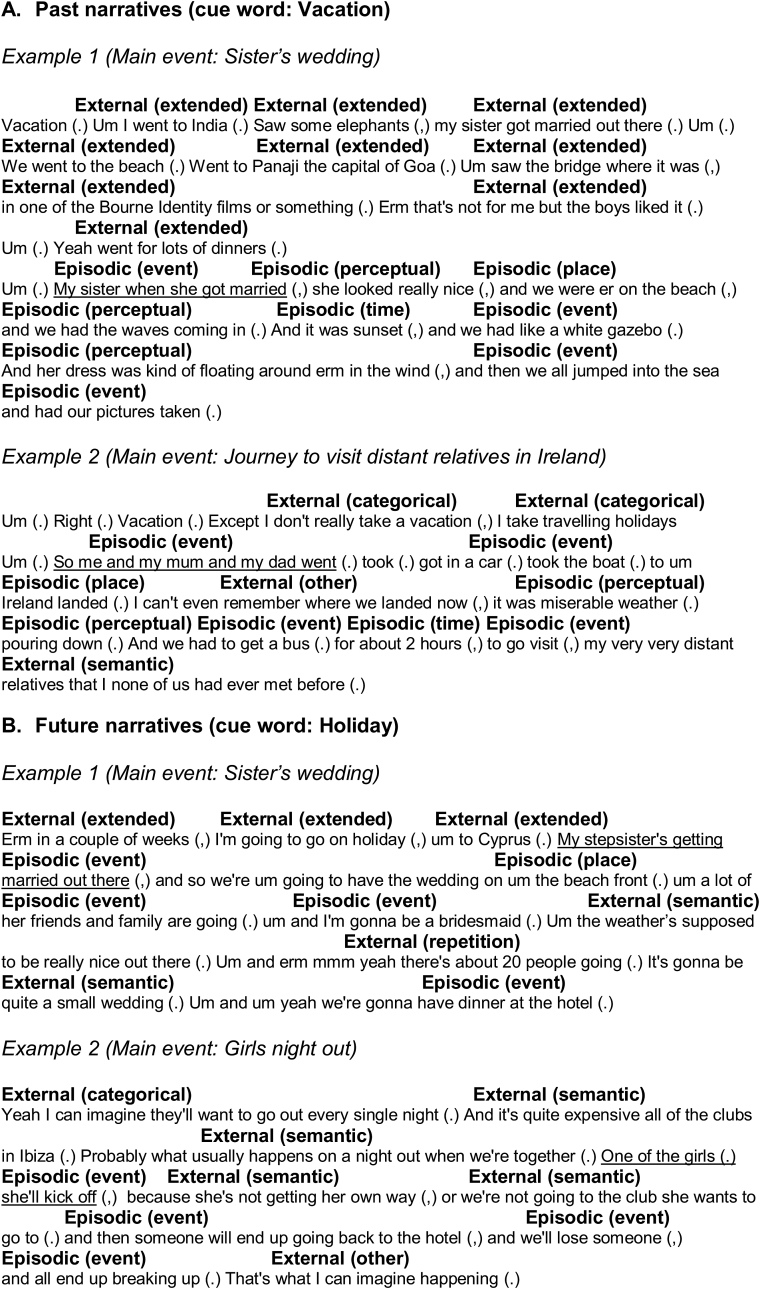
Examples of internal (episodic) and external details from past (A) and future (B) autobiographical narratives. The main event was required to be specific in time and place. If more than one specific event was provided, the event described in the most detail was coded as ‘internal’ and the other as ‘external’ (see Scoring). The main event (i.e., the event described in the most detail) is labelled for each example (e.g., ‘Sister's wedding’ for Past Example 1) and we underline the transcript to show where this event begins. The reader is referred to Levine et al. (2002, Table 1) and Hodgetts et al. (2017a, Table 1), for further details on scoring of subcomponent coding categories (subcomponent categories indicated here by bold text in brackets).

The standardized AI scoring procedure (Levine et al., 2002) was used. Events (past and future) generated were segmented into distinct chunks of information in order to allow analysis of episodic and non-episodic detail within each. These chunks were typically characterized by grammatical clauses that referenced a unique occurrence, observation or thought (Levine et al., 2002). Two broad categories were used to categorize details: ‘internal’ details (which described strictly the *main* event) and ‘external’ details (information concerning events technically external to the main event being scored, including extended events, alongside repetitions and decontextualized semantic details). As the main event was required to refer to a specific time and place, and thus can be considered ‘episodic’ (Conway, 2005; Tulving, 2002), it will be referred to as such henceforth. As in prior work (see Levine et al., 2002; Palombo et al., 2018a; Strikwerda-Brown, Mothakunnel, Hodges, Piguet, & Irish, 2019), if a participant described more than one event that was specific in time and place, the event that was described in the most precise detail was designated the main event (e.g., ‘Sister's wedding’ in Fig. 1A) and thus coded for ‘episodic’ details (the less specific or more extended in time event was then coded as ‘external’) (see Conway, 2005; Levine et al., 2002; Palombo et al., 2018a).

Episodic details included not only time and place details, but also any other episodic information (sensory details, thoughts and emotions) that were part of the central event (Levine et al., 2002). As such, after narratives were broadly segmented into ‘episodic’ and ‘external’ details (see above), ‘episodic’ details were subdivided into several subcomponents: event, time, place, perceptual and emotion/thought (see Fig. 1) (Hodgetts et al., 2017a). The ‘external’ details were then subdivided into semantic, categorical, extended, repetitions, tangential, or other (Fig. 1). Fig. 1 contains examples of external and episodic details from past and future narratives (see also Hodgetts et al., 2017a).

Consensus scoring was established based on the high inter-rater reliability from two raters who scored both the past and future events (intra-class correlation analysis, two-way random model: past (internal) r = 0.99; past (external) r = 1.0; future (internal) r = 0.78; future (external) r = 1.0). The values from one primary coder, who completed both the past and future scoring, were used in the analysis. All raters were blind to dMRI results.

For each event the numbers of episodic and external details were tallied, and the totals were then summed across the 10 events in each condition (past, future) to create episodic and external AI scores for each condition for each participant.

### 3.3 MRI data acquisition

Imaging data were acquired using a General Electric Healthcare (GE) 3-T HDx MRI system with an 8-channel receive-only head coil, at Cardiff University's Brain Research Imaging Centre (CUBRIC). A standard T1-weighted 3D FSPGR sequence (178 axial slices, 1 mm isotropic resolution, TR/TE = 7.8/3.0s, FOV = 256 × 256 × 176 mm, 256 × 256 x 176 data matrix, 20° flip angle) provided high-resolution anatomical images.

A diffusion weighted single-shot spin-echo Echo-Planar Imaging (EPI) pulse sequence was used to acquire whole-brain High Angular Resolution Diffusion Image (HARDI) data (60 contiguous slices acquired along an oblique-axial plane with 2.4 mm thickness and no gap, TE = 87 ms; voxel dimensions = 2.4 × 2.4 × 2.4 mm^3^; FOV = 23 × 23 cm^2^; 96 × 96 acquisition matrix). The acquisition was cardiac gated, with 30 isotropic directions at b = 1200 s/mm^2^. In addition, three non-diffusion weighted images were acquired with b = 0 s/mm^2^.

## 4 MRI preprocessing

### 4.1 Diffusion MRI

dMRI data were preprocessed using ExploreDTI version 4.8.3 (Leemans and Jones, 2009). Distortions resulting from Eddy currents and participant head motion were corrected. A particular issue for white matter pathways located near the ventricles (e.g., the fornix), is free water contamination from cerebrospinal fluid. This has been shown to significantly affect tract delineation (Concha et al., 2005). Thus, to correct for voxel-wise partial volume artifacts arising from free water contamination, the two-compartment ‘Free Water Elimination’ (FWE) procedure (Pasternak et al., 2009) was applied – this improves Diffusion Tensor Imaging (DTI)-based tract reconstruction and tissue specificity (Pasternak et al., 2014). Following FWE, corrected diffusion tensor indices were computed. Fractional anisotropy (FA) – a DTI-based index proposed to reflect axonal organization (Pierpaoli et al., 1996), reflects the extent to which diffusion within biological tissue is anisotropic (constrained along a single axis) (Beaulieu, 2002). FA values can range from 0 (fully isotropic) to 1 (fully anisotropic). The resulting free water corrected FA maps were inputs for the tractography analysis.

### 4.2 Tractography

Deterministic tractography was performed from all voxels based on constrained spherical deconvolution (CSD) (Jeurissen et al., 2011). CSD allows for the representation of bending/crossing/kissing fibers in individual voxels, as multiple peaks in the fiber orientation density function (fODF) can be extracted within each voxel (Dell'Acqua and Tournier, 2019). The step size was 1 mm, and the fODF amplitude threshold was 0.1. An angle threshold of 30° was used to prevent the reconstruction of anatomically implausible fibers.

To generate 3D fiber reconstructions of each tract segment, waypoint region-of-interest (ROI) gates were drawn manually onto whole-brain free water corrected FA maps. The waypoint ROIs defined the tracts based on a ‘SEED’ point and Boolean logical operations: ‘NOT’ and ‘AND’. The ‘NOT’ and ‘AND’ gates corresponded to whether tracts passing through were omitted from analyses or retained, respectively. These gates were combined to reconstruct the tracts, based on anatomical plausibility. Initially, a multiple ROI approach was applied to reconstruct the fornix (see Hodgetts et al., 2017a; Metzler-Baddeley, Jones, Belaroussi, Aggleton and O'Sullivan, 2011), and subsequently fornix tract subdivision was performed following the Christiansen et al. (2016) protocol.

### 4.3 Fornix reconstruction

**Fig. 2:**
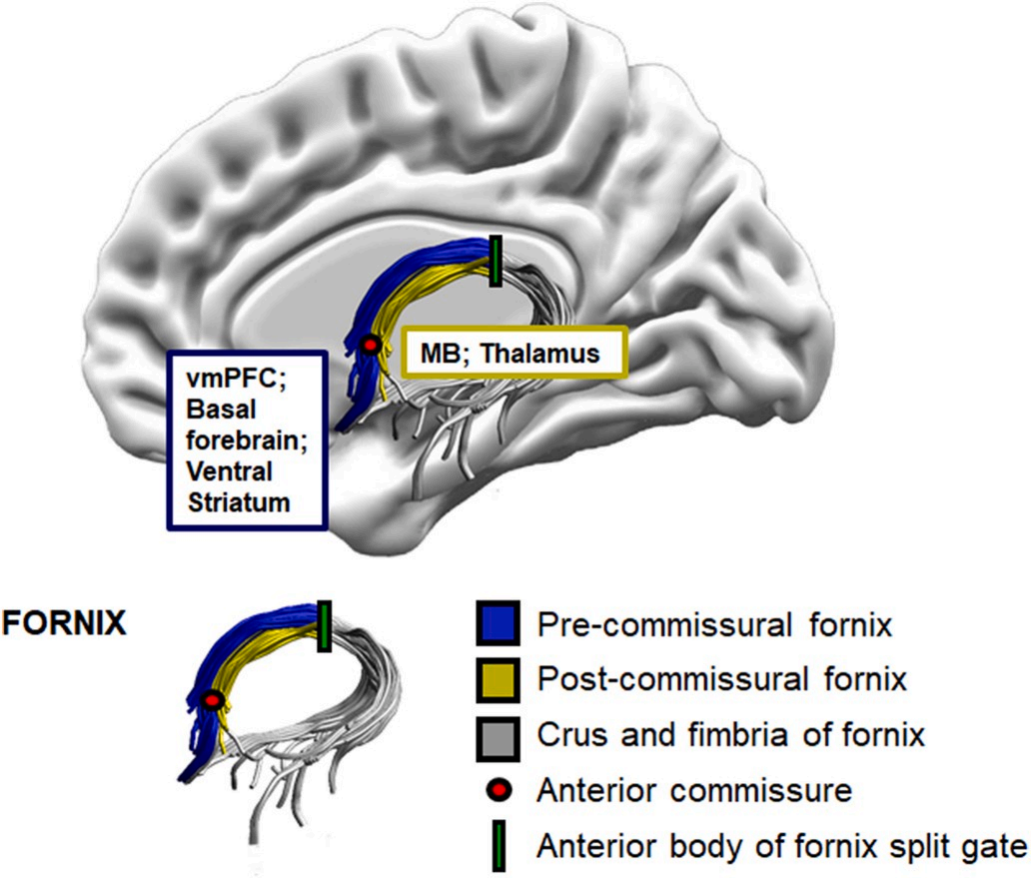
Schematic illustration of the anatomical landmarks for fornix tract subdivision, and the connecting areas of interest. vmPFC = Ventromedial Prefrontal Cortex; MB = Mammillary Bodies.

A ‘SEED’ point ROI was placed on the coronal plane, encompassing the body of the fornix. An ‘AND’ ROI was placed on the axial plane, capturing the crus fornici in both hemispheres at the lower part of the splenium of the corpus callosum. ‘NOT’ ROIs were placed intersecting the corpus callosum on the axial plane, and anterior to the fornix pillars and posterior to the crus fornici on the coronal plane. Further ‘NOT’ way-gates were placed after the initial reconstruction and ensuing visual inspection, to remove anatomically implausible fibers. Subsequently, the anterior body of the fornix was split into the pre- and post-commissural column segments (Fig. 2).

**Fig. 3:**
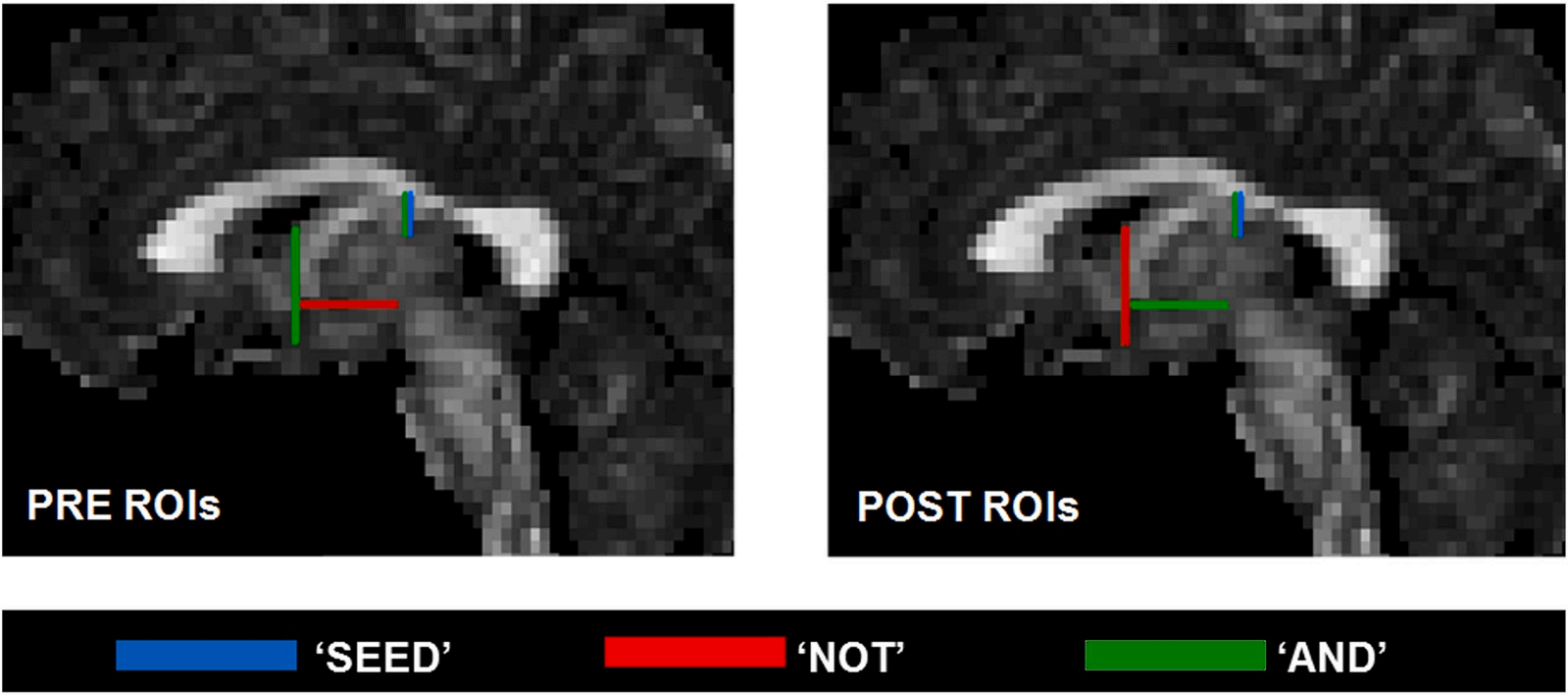
Waypoint region-of-interest (ROI) gates used for reconstructing the pre- and post-commissural fornix tract segments (Blue = SEED, Red = NOT, Green = AND). (For interpretation of the references to colour in this figure legend, the reader is referred to the Web version of this article.)

**Fig. 4:**
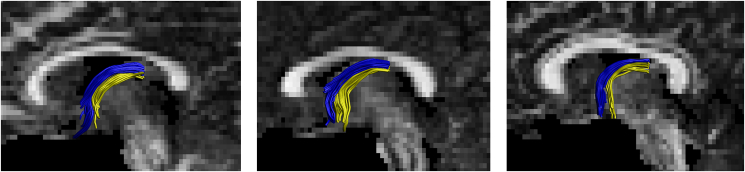
Example reconstructions for the pre- and post-commissural fornix segments (Blue = Pre, Yellow = Post). (For interpretation of the references to colour in this figure legend, the reader is referred to the Web version of this article.)

Waypoint ROIs for the pre-post split (Fig. 3) were based on the protocol described in Christiansen et al. (2016), and example tract reconstructions are depicted in Fig. 4. After tract reconstruction for each participant, mean FA values were calculated by averaging the values at each 1 mm step along each segment.

### 4.4 Pre- and post-commissural fornix reconstruction

The fornix was split, isolating the anterior-body, by an ‘AND’ gate positioned at the point of the downward bend to the crus and fimbria of the fornix. In line with Christiansen et al. (2016), fibers of the crus and fimbria of the fornix were excluded from the anterior-body and hence pre- and post-commissural fornix reconstructions. Partial volume effects due to the intermingling of the two fiber populations beyond the crus were, therefore, minimized (Saunders and Aggleton, 2007). In addition, this procedure avoided ‘jumping’ where tract voxels that pass close to, or across, neighboring tract voxels ‘jump’ onto them (Jones and Cercignani, 2010). This split was conducted using the tract segmentation tool “splitter” within ExploreDTI version 4.8.3.

The anterior-body of the fornix was then divided into the pre- and post-commissural segments. This delineation took advantage of the manner in which the fibers separate at the anterior columns of the fornix. At this level, the segments contain approximately the same number of fibers (Powell et al., 1957). The pre-commissural fornix was delineated by positioning an additional ‘AND’ gate on the coronal plane at the anterior-commissure, as well as an additional ‘NOT’ gate meeting this ‘AND’ gate on the axial plane. For the post-commissural fornix reconstruction, the additional ‘NOT’ and ‘AND’ gates placed for reconstruction of the pre-commissural fornix were swapped (see Fig. 3). Thus, for the pre-commissural fornix, tracts were included only if they extended anterior to the anterior commissure, and for the post-commissural fornix only tracts running posterior to the anterior commissure were retained (see Fig. 4; Christiansen et al., 2016).

### 4.5 Grey matter volumetrics

T1-weighted images were corrected for spatial intensity variations using FMRIB's Automated Segmentation Tool (FAST; Zhang et al., 2001). Bilateral grey matter volumes (expressed as a proportion of estimated total intracranial volume) of the hippocampus were subsequently obtained using FMRIB's Integrated Registration & Segmentation Tool (FIRST; Patenaude et al., 2011). Volumes for the vmPFC ROI were derived using FreeSurfer (surfer.nmr.mgh.harvard.edu: Destrieux et al., 2010), via summing volumes of the medial orbitofrontal cortex (mOFC) and rostral anterior cingulate cortex (rACC) parcels. One participant was removed from the grey matter analyses due to poor overall data quality on the T1 FSPGR.

### 4.6 Statistical analysis

As higher values of FA are considered indicative of increased myelination and improved organization, cohesion, and compactness of white matter fiber tracts (Beaulieu, 2002), we predicted a positive association between pre-commissural FA and the episodic richness of past and future constructions. Thus, directional Pearson's correlations were conducted between individual's total scores of episodic and external details produced for the ten past and future narratives; and individual's episodic past and future scores and their FA values for the pre- and post-commissural fornix (Lakens, 2016). Vovk-Sellke Maximum *p* –ratios (VS-MPR) were computed: based on the *p* -value, the maximum possible odds in favor of H₁ over H₀ equals 1/(-*e p* log(*p*)) for *p* ≤ 0.37, where log is the natural logarithm and *e* is its constant base (Benjamin and Berger, 2019). The VS-MPR represents the largest odds in favor of the alternative hypothesis relative to the null hypothesis that is consistent with the observed data, aiding the interpretation of p-values (Benjamin and Berger, 2019). Complementary non-parametric Spearman's rho rank tests were also conducted for the key correlations. These are less sensitive to potential outliers and differences in range (Croux and Dehon, 2010). In addition, partial correlations were conducted for the key episodic-fornix microstructure correlations, to control for the contribution of the number of external details given, verbal fluency (see below) and regional grey matter volumes. Analyses were conducted in JASP (2018, version 0.9.1.0) and RStudio (2015).

## 5 Results

### 5.1 Correlations between tract microstructure and past-future AI scores

#### 5.1.1 Number of details produced (episodic and external) for the past and future narratives

**Fig. 5:**
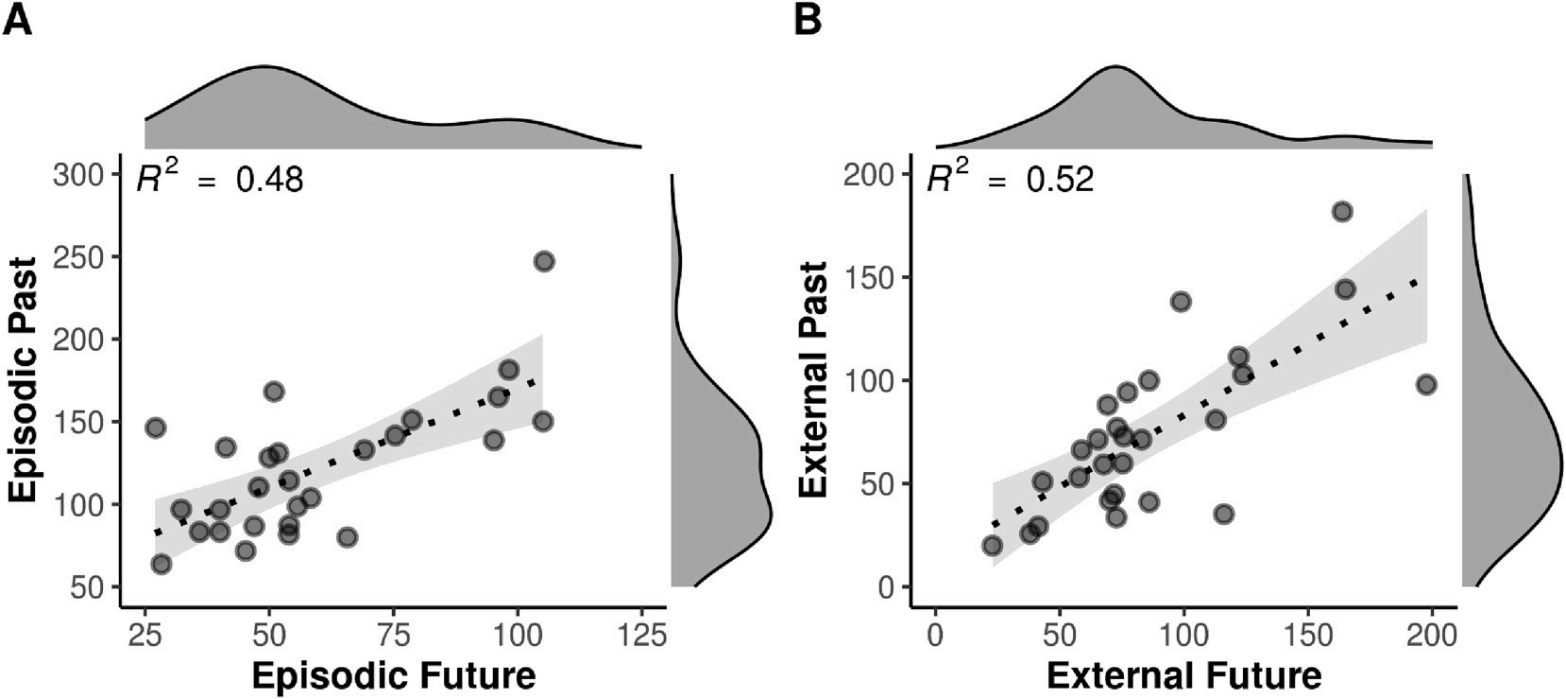
**(A, B)**. Scatterplots depicting correlations between the number of details produced for the past versus the future AI narratives (A. Episodic, B. External) (*N* = 27). Marginal density is displayed on the opposite axis. Grey shading equals the 95% CI.

Consistent with previous studies (e.g. Addis et al., 2009b; Addis et al., 2008; Race et al., 2011), the total number of episodic details (summed across the 10 cue words) an individual recalled for the past (mean = 121.3, median = 114, SD = 40.8, range = 64–247) correlated strongly with the number of episodic details imagined for the future (mean = 59.3, median = 54, SD = 23.4, range = 27–105) (Fig. 5A. Pearson's r = 0.69, p < 0.001, VS-MPR = 1027.33). Additionally, in line with previous studies, there were significantly more episodic details given for the past in comparison to the future (*t* (26) = 10.75, p < 0.001, Cohen's d_z_ = 2.07, paired *t*-test). The number of external details an individual recalled for the past (mean = 73.8, median = 71, SD = 39, range = 20–182) also correlated significantly with the number of external details imagined for the future (mean = 86.5, median = 75, SD = 40.8, range = 23–198) (Fig. 5B. Pearson's r = 0.73, p < 0.001, VS-MPR = 3254.64). There were also significantly more external details given for the future in comparison to the past (*t* (26) = 2.23, p = 0.035, d_z_ = 0.43, paired *t*-test), again consistent with previous findings (see Irish and Piguet, 2013 for discussion). The number of episodic details an individual recalled for the past also correlated with the number of external details recalled for the past (Pearson's r = 0.35, p = 0.035, VS-MPR = 3.15); this was not the case, however, for the future (Pearson's r = −0.16, p = 0.783, VS-MPR = 1.00).

#### 5.1.2 Episodic past details and pre-/post-commissural fornix FA

**Fig. 6:**
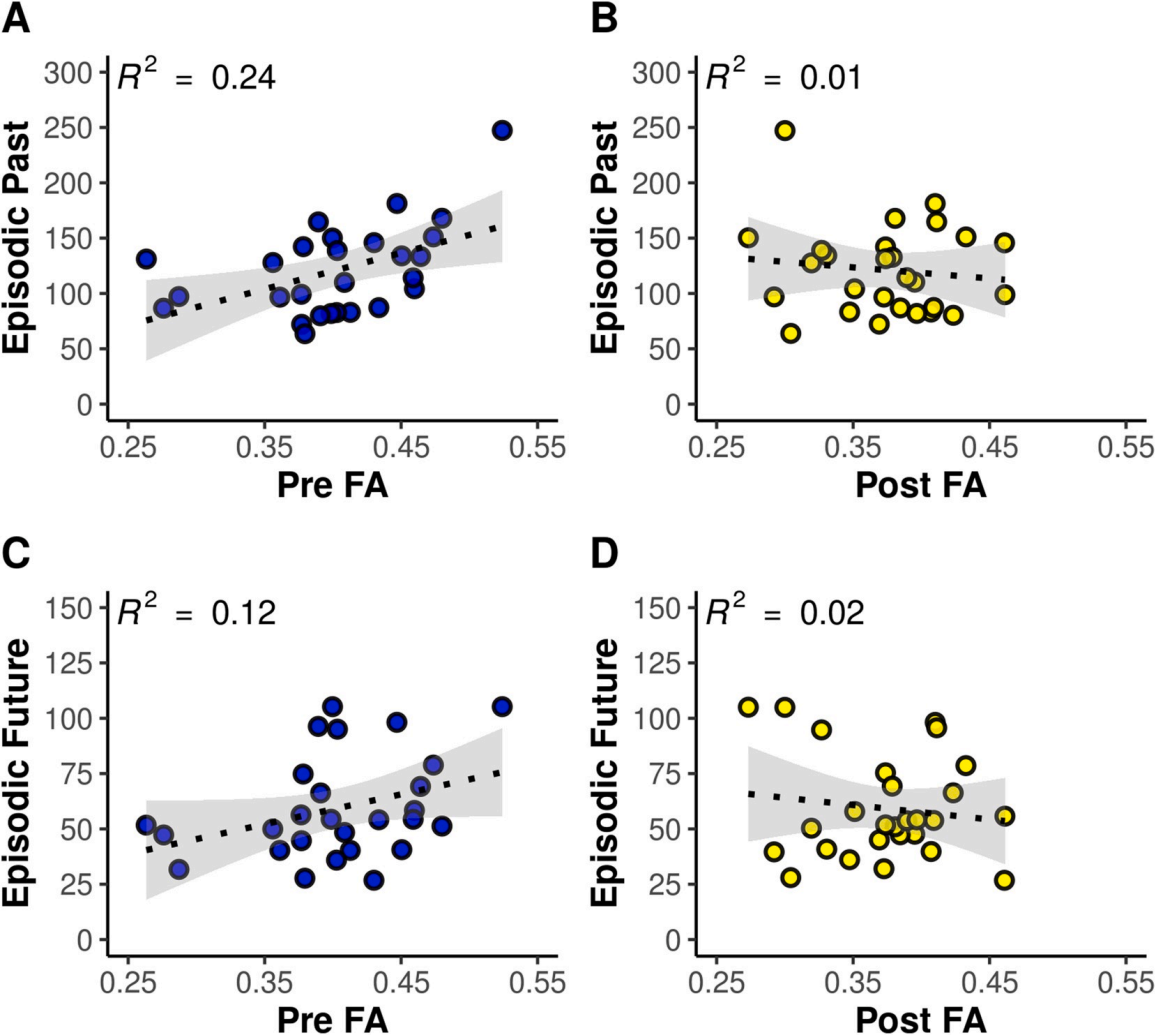
**(A**–**D)**. Scatterplots depicting the correlations of episodic past (A, B) and future (C, D) AI details with pre-/post-commissural fornix microstructure (fractional anisotropy, FA). Number of episodic past/future details (summed over 10 cue words) is plotted on the y-axis (*N* = 27). Grey shading equals the 95% CI.

We found a significant positive correlation between the number of episodic past details and pre-commissural fornix FA (Fig. 6A. Pearson's r = 0.49, p = 0.005, VS-MPR = 14.49, Spearman's rho = 0.464, p = 0.007, VS-MPR = 10.09). There was no significant correlation between post-commissural fornix FA and episodic past details (Fig. 6B. Pearson's r = −0.12, p = 0.725, VS-MPR = 1.00, Spearman's rho = 0.02, p = 0.457, VS-MPR = 1.00). There was no significant correlation between pre-commissural fornix FA and post-commissural fornix FA (Pearson's r = 0.03, p = 0.440, VS-MPR = 1.00). The correlation between episodic past details and pre-commissural fornix FA was significantly greater than between episodic past details and post-commissural fornix FA (Steiger z (27) = 2.29, p = 0.011) (computed using R package ‘cocor’, Diedenhofen and Musch, 2015).

The correlation between episodic past details and pre-commissural fornix FA was also significantly greater than between external past details and pre-commissural fornix FA (Steiger z (27) = 1.69, p = 0.046). Additionally, when controlling for the number of external details produced by the individual, the correlation between episodic past details and pre-commissural fornix FA remained significant (Pearson's r_partial_ = 0.48, p = 0.007, Spearman's rho_partial_ = 0.47, p = 0.007).

#### 5.1.3 Episodic future details and pre-/post-commissural fornix FA

The findings for the episodic future simulation details mirrored those for episodic past retrieval. There was a significant positive correlation between the total number of episodic future details (summed over the 10 cue words) and pre-commissural fornix FA (Fig. 6C. Pearson's r = 0.35, p = 0.035, VS-MPR = 3.11, Spearman's rho = 0.33, p = 0.045, VS-MPR = 2.62), and, correspondingly, there was no significant correlation between episodic future details and post-commissural fornix FA (Fig. 6D. Pearson's r = −0.14, p = 0.752, VS-MPR = 1.00, Spearman's rho = 0.09, p = 0.330, VS-MPR = 1.01). The correlation between episodic future details and pre-commissural fornix FA was also significantly greater than between episodic future details and post-commissural fornix FA (Steiger z (27) = 1.78, p = 0.038). The correlation between episodic future details and pre-commissural fornix FA was not significantly greater than between external future details and pre-commissural fornix FA, however, when controlling for the number of external details generated, the correlation between episodic future details and pre-commissural fornix FA remained significant (Pearson's r_partial_ = 0.38, p = 0.028, Spearman's rho_partial_ = 0.33, p = 0.0499). In addition, the correlation between episodic past details and pre-commissural fornix FA was not significantly greater than that observed between episodic future details and pre-commissural fornix FA (Steiger z (27) = 0.96, p = 0.169).

### 5.2 Influence of grey matter volume

When hippocampal and vmPFC volume was controlled for, the correlation between episodic past details and pre-commissural fornix FA remained significant (Pearson's r_partial_ = 0.54, p = 0.003), and there was no significant association between post-commissural fornix FA and episodic past details (Pearson's r_partial_ = −0.18, p = 0.200). Likewise, the correlation between episodic future details and pre-commissural fornix FA remained significant when controlling for hippocampal and vmPFC volume (Pearson's r_partial_ = 0.40, p = 0.027), and there was no significant association between post-commissural fornix FA and episodic future details (Pearson's r_partial_ = −0.02, p = 0.471).

### 5.3 Post-hoc analysis: influence of verbal fluency

Similarities between remembering the past and imagining the future might reflect the influence of general, non-episodic processes, such as verbal fluency and narrative style (Addis and Schacter, 2012). Our participants also completed a measure of semantic verbal fluency (‘category fluency’, as derived from the Delis-Kaplan Executive Function battery; (Ardila et al., 2006; Delis et al., 2001). For this test, participants had 1 min to generate as many unique exemplars as possible for the category ‘Animals’ (mean = 13.41, SD = 2.17). We found that the correlation between the episodic content of past and future scenarios remained significant when controlling for verbal fluency (Pearson's r_partial_ = 0.70, p < 0.001), as did the correlation between pre-commissural fornix FA and both past (Pearson's r_partial_ = 0.50, p = 0.004) and future (Pearson's r_partial_ = 0.35, p = 0.039) episodic details.

## 6 Discussion

Neuropsychological and fMRI studies, founded on Tulving's observations that amnesic individual K.C. could no more imagine his future than he could recollect his past (Tulving, 1985; see Gao et al., 2020 for neuropathological findings in KC), suggest that the ability to vividly remember past episodes and imagine future ones involves two closely connected regions: hippocampus and vmPFC (McCormick et al., 2018; Schacter et al., 2017). Despite evidence of a direct connection from hippocampus to vmPFC mediated by the pre-commissural fornix (Aggleton et al., 2015), it is unknown whether this connectivity supports both past and future-oriented episodic thinking.

To address this, we applied a novel anatomically-guided protocol that allows the pre-commissural and post-commissural fornix fibers to be separately reconstructed *in vivo* (Christiansen et al., 2016). To assess both past- and future-oriented thinking, we used an adapted autobiographical cueing paradigm (Cole et al., 2012; Crovitz and Schiffman, 1974) alongside a validated coding scheme that specifically parses episodic from non-episodic detail within individuals' real-world descriptions (Levine et al., 2002). Using this approach, we found that inter-individual variation in *pre*-commissural, but not *post*-commissural, fornix microstructure was significantly correlated with the amount of ‘internal’ episodic detail produced during the construction of both past and future events. These findings deepen our understanding of hippocampal-vmPFC interactions in human episodic autobiographical memory and future thinking and provide a ‘structural realization’ of hippocampal-vmPFC functional connectivity (Kosslyn and Van Kleeck, 1990), that is, a direct relationship between the microstructure of the fiber pathway connecting these distributed regions and individual differences in the episodic content of past and future thinking.

Notably, the link between pre-commissural fornix FA and the episodic detail of past and future constructions held when controlling for ‘external’ content, which is to a significant extent but not exclusively semantic in nature, such as related facts, alongside reflections on the meaning of what happened, or off-topic commentary (Levine et al., 2002; Renoult et al., 2020; Strikwerda-Brown et al., 2019). This concurs with findings that the non-episodic content of past and future narratives is unaffected in patients with lesions to the hippocampus (Race et al., 2011) and vmPFC (Bertossi et al., 2016). Such “converging dissociations” (Nyberg and Tulving, 1996) provide additional support for Tulving's (e.g. Tulving, 1983, 2002) claim that episodic and semantic memory are distinct (albeit highly interacting) neurocognitive systems (see Renoult and Rugg, 2020, for an historical perspective on Tulving's episodic-semantic dichotomy; and Renoult et al., 2019, for an update on the episodic-semantic distinction). Such findings also build upon previous work that reported a double dissociation in the white matter correlates of episodic and semantic autobiographical memory (Hodgetts et al., 2017a; but see Memel et al., 2020, for a failure to replicate this double dissociation; and see Murray et al., 2017, for an alternative theoretical account of these dissociations).

Our findings highlight the importance of hippocampus-vmPFC structural connectivity mediated by the pre-commissural fornix (Aggleton et al., 2015; Cenquizca and Swanson, 2007), in episodic construction across past and future events. This builds upon previous fMRI studies that have shown that functional coupling between these distributed regions is increased during both the retrieval of autobiographical memories and the construction of future events (Campbell et al., 2018; McCormick et al., 2015). One recent study, which used structural equation modeling of fMRI data, found increased functional connectivity from anterior hippocampus *to* vmPFC when participants retrieved autobiographical memories in response to cue words (McCormick et al., 2015). Similarly, another investigation applied dynamic causal modeling to fMRI data and found that anterior hippocampus *to* vmPFC effective connectivity increased specifically during the initial construction of episodic future events (Campbell et al., 2018). From this, the authors proposed that the hippocampus initiates event construction in response to retrieval cues, which then drives activation in the vmPFC where episodic details may be further integrated.

This conceptualization is consistent with previous work in both humans and rodents that demonstrated that hippocampal activity precedes medial PFC activity during memory retrieval (McCormick et al., 2015; Place et al., 2016), and with findings in rodents that hippocampus mediates theta drive to vmPFC (O'Neill et al., 2013). Optogenetic studies in mice (e.g. Ciocchi et al., 2015) have also shown that during memory retrieval ventral hippocampal signals carrying contextual information are sent directly to medial PFC, facilitating coordinated activity between these areas.

The differential contributions of the hippocampus and vmPFC to episodic constructive processes are hotly debated (McCormick et al., 2018; Robin and Moscovitch, 2017; Schacter et al., 2017). According to scene construction theory, the hippocampus, and particularly the subiculum, plays a central role in forming representations of spatially coherent scenes across memory, perception and imagination (Gaffan, 1991; Hodgetts et al., 2017b; Zeidman and Maguire, 2016), and these conjunctive scene representations have been proposed to provide a scaffold when constructing both past and future events (Barry and Maguire, 2019; Murray et al., 2017; Robin, 2018). In contrast, the constructive episodic simulation hypothesis contends that the construction of spatiotemporal contexts arises out of a more general relational processing mechanism (Eichenbaum and Cohen, 2014) housed in anterior hippocampus, which is also responsible for the integration of other event details into the event representation (Addis, 2018; Addis and Schacter, 2012; Schacter et al., 2012; see also Rosenbaum et al., 2009; and Sheldon and Levine, 2016).

The vmPFC's contribution to episodic construction, by contrast, has been linked to demands on schematic representations (Gilboa and Marlatte, 2017; Robin and Moscovitch, 2017; Sheldon and Levine, 2016; van Kesteren et al., 2012), in particular the self-schema (Buckner and Carroll, 2007; D'Argembeau, 2013). For instance, Kurczek et al., 2015; see also Verfaellie et al., 2019) compared the number of references to ‘the self’ included in autobiographical event narratives from patients with bilateral hippocampal or medial PFC damage as well as healthy controls. Patients with medial PFC damage, despite being able to construct highly detailed episodic events, produced relatively few self-references, and they incorporated themselves in the narratives of their (re)constructions less frequently than the healthy participants. Patients with hippocampal damage showed the opposite pattern: they were impaired in their ability to construct highly detailed episodic events across time periods but not in their incorporation of the self. Building on the ideas of Wheeler et al. (1997), and in line with Tulving’s (2005) emphasis on the importance of the self to episodic memory, we have previously suggested (Murray et al., 2017) that hippocampal-vmPFC connectivity serves to (re)create complex conjunctive representations in which one's self is oriented in a particular time, place, and overall situational context (Murray et al., 2017). These conjunctive representations may subsequently constrain further retrieval and construction by the hippocampus (Campbell et al., 2018; Graham et al., 2010; Place et al., 2016; Preston and Eichenbaum, 2013). Thus, recall/imagination of autobiographical episodes involves a prefrontal ‘self’ system that can work in conjunction with the MTL system to help individuals recombine episodic details to construct a *personally relevant* past/future event (Tulving, 2005; Wheeler et al., 1997; see also Buckner and Carroll, 2007; Karapanagiotidis et al., 2017; but see scene construction theory - Barry and Maguire, 2019; Ciaramelli et al., 2019; McCormick et al., 2018 - for an alternative account of vmPFC contributions that de-emphasizes self-processes).

Critically, the pre-commissural fornix does not carry reciprocal projections from the vmPFC to the hippocampus (which are indirect via the thalamic nucleus reuniens and entorhinal cortex) (Aggleton et al., 2010; Murray et al., 2017; Preston and Eichenbaum, 2013), but only carries connections to the vmPFC from the hippocampus (primarily subiculum/CA1) (Aggleton et al., 2015; Cenquizca and Swanson, 2007). While several models of episodic memory emphasize the importance of bi-directional interactions between hippocampus and vmPFC (e.g. Eichenbaum, 2017; Preston and Eichenbaum, 2013; Robin and Moscovitch, 2017; Sheldon and Levine, 2016), with vmPFC playing a regulatory (Barry and Maguire, 2019; Eichenbaum, 2017; Preston and Eichenbaum, 2013; Robin and Moscovitch, 2017) or even initiating (Barry et al., 2019; McCormick et al., 2018) role in episodic construction, our findings reveal that the direct inputs that the hippocampus provides to vmPFC are important for individual differences in episodic memory and future thinking, and that the pre-commissural fornix is a key link in this broader hippocampal-vmPFC circuit.

Whilst there are strong parallels between past and future episodic thinking at the individual level, this is not to say there are no differences between remembering and imagining. In particular while the correlation between episodic past details and pre-commissural fornix FA was not significantly greater than that observed between episodic future details and pre-commissural fornix FA, the evidence in support of the former - as indexed by the VS-MPR (Benjamin and Berger, 2019) - was stronger than for the latter. This likely reflects the fact that, as in other studies (e.g. Addis et al., 2009b; Addis et al., 2008; Bertossi et al., 2016; Race et al., 2011) individuals represented past events in greater specific detail than they represented future events, and relied more heavily on semantic knowledge to frame or scaffold imagined than remembered events (D'Argembeau and Mathy, 2011; Irish and Piguet, 2013).

While our findings highlight a key role for hippocampal structural connectivity with medial PFC in constructing self-relevant event representations, previous work in humans, primates and rodents has tended to emphasize the importance of direct connectivity between the hippocampus and medial diencephalon (i.e., mammillary bodies and thalamus) in spatial and contextual memory (Aggleton and Brown, 1999; Aggleton et al., 2008; Parker and Gaffan, 1997; Rosenbaum et al., 2014), connectivity which is mediated by the *post*- but not the *pre*-commissural fornix (Aggleton et al., 2010; Christiansen et al., 2016; Mathiasen et al., 2019; Vann and Nelson, 2015). While the current findings seemingly challenge this account, one caveat is that our post-commissural fornix tract reconstructions principally involve the connections of the hippocampus with the hypothalamus, including the mammillary bodies, and largely exclude the projections to the anterior thalamic nuclei, as these turn towards posterior regions as the fornix columns descend (Aggleton et al., 2010; Christiansen et al., 2016; Poletti and Creswell, 1977). These thalamic fibers do not form a discrete tract, rather they remain diffuse (Mathiasen et al., 2019). While previous work has demonstrated that thalamic degeneration can impair both episodic autobiographical memory and future thinking (Irish et al., 2013; Rosenbaum et al., 2014), there are, however, several *non-fornical* connections between the hippocampal formation and the anterior thalamic nuclei that may be critical to episodic memory (Aggleton et al., 2010; Bubb et al., 2017).

Strikingly, and in line with our findings, Vann and colleagues (Vann, 2013; Vann, Erichsen, O'Mara and Aggleton, 2011; Vann and Nelson, 2015) have reported that selective lesions to the descending post-commissural fornix columns in rats, which disconnect the subicular projections to the mammillary bodies (but leave intact hippocampal connections with the anterior thalamic nuclei), have little if no impact on spatial memory tests that are sensitive to mammillary body, mammillothalamic tract, anterior thalamic, and hippocampal lesions. One implication of these findings (with the caveat that they represent a single dissociation) is that the direct hippocampal-mammillary connectivity mediated by the post-commissural fornix may be less critical than the direct hippocampal-vmPFC connectivity mediated by the pre-commissural fornix to certain episodic memory tasks including (as here) those that place demand on constructive and self-referential processing (see also Tedder et al., 2016).

The present study has limitations that should be addressed in future studies. Similarities between remembering the past and imagining the future could potentially reflect individual differences in non-episodic processes such as narrative style (Gaesser et al., 2011) and verbal fluency (Addis and Schacter, 2012). Our findings, however, held when controlling for verbal fluency. Further, previous studies in individuals with hippocampal (Race et al., 2011) and vmPFC (Bertossi et al., 2017) damage show that general narrative abilities (measured by a picture description task) cannot account for deficits in episodic memory and future thinking. Nevertheless, future individual difference studies could incorporate additional measures of such non-episodic abilities. The field would greatly benefit from the development of nonverbal measures of episodic memory and future thinking (Wilkins and Clayton, 2019).

Whilst we strived to ensure that participants constructed novel future events in response to cue-words, it is possible that some responses reflect a ‘recasting’ of entire past events as future events (Addis and Schacter, 2012). Replicating our findings using an experimental recombination paradigm, in which participants are required to recombine episodic details extracted from their own past events (Addis et al., 2009a), would address this issue.

Although FA is highly sensitive to the microstructure of fibers, it lacks biological specificity, and may reflect myelination, axon diameter and packing density, axon permeability and fiber geometry (Jones et al., 2013). Concha, Livy, Beaulieu, Wheatley, and Gross (2010), using human DTI-histology comparisons, found that FA of the fornix was strongly positively correlated with axonal membranes (cumulative membrane circumference) and axonal density. Variation in such microstructural properties can influence communication efficiency and synchronicity between distal brain regions (Jbabdi and Behrens, 2013; Pajevic et al., 2014). Future studies using multi-shell diffusion MRI and advanced biophysical modeling to estimate specific microstructural properties including axon density (Assaf et al., 2017) will provide further insight into the specific biological attributes underlying these microstructure-cognition associations.

Further, while our sample size was comparable to related investigations (e.g. Palombo et al., 2018a; Postans et al., 2014), replicable and precise results are more likely when statistical power is high (Button et al., 2013; Yarkoni, 2009). Critically, however, it is entirely possible for low-power experiments to have high evidential value, and for high-power experiments to have low evidential value (Dienes and Mclatchie, 2018; Wagenmakers et al., 2015). To assess the extent to which a particular data set provides evidence for or against the null hypothesis, it is recommended that researchers use likelihood ratios or Bayes factors (Benjamin and Berger, 2019; Dienes and Mclatchie, 2018; Wagenmakers et al., 2015). Here, VS-MPRs (the largest odds in favor of the alternative hypothesis relative to the null hypothesis that is consistent with the observed data) showed that our findings provide a good level of diagnosticity (Benjamin and Berger, 2019), especially for the correlation between episodic past details and pre-commissural fornix FA. Most importantly, Memel et al. (2020) have directly replicated our previously published finding from this sample (Hodgetts et al., 2017a) of a significant positive correlation between FA of the fornix as a unified bundle and the episodic (especially spatiotemporal) detail of autobiographical memories as scored using the AI protocol. Finally, the fact that findings converge across different methodologies (dMRI, fMRI, neuropsychology) provides confidence in their robustness (Nyberg and Tulving, 1996). That said, it will be important to extend our findings to larger lifespan samples (Kernbach et al., 2018).

In summary, we report a novel association between white matter microstructure of the pre-commissural fornix and episodic past and future thinking, thus elucidating a potential anatomical mechanism by which direct hippocampal-to-vmPFC connectivity supports constructive episodic processing. These findings provide important support for the idea of a ‘core’ network supporting both the re-construction of autobiographical events and the construction of hypothetical personal future events, and that individual differences in structural connectivity may reflect how richly people can “mentally roam at will over what has happened, as readily as over what might happen” (Tulving, 2002).

## CRediT authorship contribution statement

**Angharad N. Williams:** Conceptualization, Formal analysis, Data curation, Visualization, Writing - original draft. **Samuel Ridgeway:** Conceptualization, Formal analysis, Data curation, Writing - review & editing. **Mark Postans:** Investigation, Data curation, Writing - review & editing. **Kim S. Graham:** Conceptualization, Funding acquisition, Supervision, Writing - review & editing. **Andrew D. Lawrence:** Conceptualization, Funding acquisition, Supervision, Writing - review & editing. **Carl J. Hodgetts:** Conceptualization, Supervision, Writing - review & editing.

## Declaration of competing interest

The authors declare no competing financial and non-financial interests.
